# Long-term mechanical ventilation: State of the evidence and future directions

**DOI:** 10.1177/14799731231199764

**Published:** 2023-08-31

**Authors:** Sunita Mathur, Michael Steiner

**Affiliations:** 1 School of Rehabilitation Therapy, 4257 Queen’s University, Kingston, ON, Canada; 2 Respiratory Medicine, 4488 University of Leicester, Leicester, UK

The series on long-term ventilation is comprised of three narrative reviews, on the topics of respiratory therapies for people with neuromuscular disease, specifically amyotrophic lateral sclerosis (ALS),^1^ transitions in care for ventilator-assisted individuals,^2^ and palliation and end of life care in neuromuscular disorders.^3^ This series highlights current practices and optimal collaborative care plans for ventilator-assisted individuals, current evidence and avenues for research directions in the area of long-term ventilation.

Sales de Campos et al.^1^ provide a comprehensive review of respiratory therapies for individuals with ALS, including non-invasive ventilation (NIV). The benefits of NIV are discussed including improved survival and quality of life. The authors also emphasize that the choice for initiating or discontinuing NIV lies with the patient, and their caregivers.

The review on transitions in care by Xioa et al.^2^ highlights the complexities of three major transitions: hospital to home; pediatric to adult care; and active treatment to end of life care. The authors discuss the need for interprofessional collaborative care, and the negative consequences of a suboptimal transition. The authors also describe formal transition programs and how these may lead to better outcomes in the transition process. The role of caregivers and the burden that they face in transitions in care is discussed as a key part of the transition process.

Managing caregiver burden was also discussed in the context of palliative care,^3^ and the increasing burden faced by caregivers when the health of their family member deteriorates. The importance of the healthcare team in supporting caregivers, including respite care, is emphasized as part of end of life care support.

One key issue that is highlighted in the reviews is the impact that COVID-19 had on the care of ventilator-assisted individuals. Xioa et al.^2^ described the greater use of tele-medicine, and remote monitoring for facilitating transitions in care during the pandemic. Developments in ventilator technology have opened the opportunity for remote monitoring of home ventilation and whilst this may enhance the effectiveness and quality assurance of home therapy, there is a risk of distancing the patient from human support from the homecare team and widening inequality because of the digital divide. It may, however, lead to more cost-effective, and smooth transition process and can be explored further as part of transition models. Sales de Campos et al.^1^ describe how there has been a shift towards less reliance on pulmonary function testing during the pandemic, in the initiation and monitoring of NIV. Elverson et al.^3^ described how the pandemic highlighted inequities in access to palliative care, especially for individuals with neuromuscular disorders and malignancies. This may lead to improved access to end of life care in equity seeking groups.

The initiation, ongoing support and discontinuation of long term mechanical ventilation is a complex process which requires collaborative care among the ventilator-assisted individual, their caregivers and interprofessional healthcare team. As the availability of home ventilatory support widens it is likely that increasing numbers of people will be reliant on such therapy. This series provides a timely summary of current, evidence-based practice for ventilator-assisted individuals and emerging issues that require further research.

## References

[1] Sales de Campos P Olsen WL Wymer JP , et al. Respiratory therapies for amyotrophic lateral sclerosis: a state of the art review. Chron Respir Dis 2023; 20: 14799731231175915. 37219417 10.1177/14799731231175915 PMC10214054

[2] Xiao L Amin R Nonoyama ML . Long-term mechanical ventilation and transitions in care: a narrative review. Chron Respir Dis 2023; 20: 14799731231176301. 37170874 10.1177/14799731231176301 PMC10184211

[3] Elverson J Evans H Dewhurst F . Palliation, end of life care and ventilation withdrawal in neuromuscular disorders. Chron Respir Dis 2023; 20: 14799731231175911. 37199317 10.1177/14799731231175911 PMC10201157

